# Phytochemical Profile and In Vitro Antioxidant and Photobiological Properties of Different Extracts from *Prangos ferulacea* Lindl.

**DOI:** 10.3390/antiox12020384

**Published:** 2023-02-05

**Authors:** Mariangela Marrelli, Francesca Giordano, Maria Rosaria Perri, Valentina Amodeo, Noemi Baldino, Carmine Lupia, Dimitar Uzunov, Vincenzo Musolino, Filomena Conforti, Maria Luisa Panno

**Affiliations:** 1 Department of Pharmacy, Health and Nutritional Sciences, University of Calabria, 87036 Rende, Italy; 2 Department of Information, Modeling, Electronics and System Engineering (D.I.M.E.S.), University of Calabria, 87036 Rende, Italy; 3 Mediterranean Etnobotanical Conservatory, Sersale, 88054 Catanzaro, Italy; 4 National Etnobotanical Conservatory, Castelluccio Superiore, 85040 Potenza, Italy; 5 National Museum of Natural History, Bulgarian Academy of Sciences, 1000 Sofia, Bulgaria; 6 Laboratory of Pharmaceutical Biology, Department of Health Sciences, Institute of Research for Food Safety & Health (IRC-FSH), University of Catanzaro Magna Græcia, 88100 Catanzaro, Italy

**Keywords:** Apiaceae, *Cachrys*, furanocoumarins, melanoma, photosensitizing agents, plant extracts, *Prangos*

## Abstract

Interesting photobiological properties have been demonstrated for some *Cachrys* species, including *C. libanotis* L., *C. sicula* L., and *C. pungens* Jan. The present study was designed to assess the photocytotoxic activity of *Prangos ferulacea* Lindl. (synonym of *C. ferulacea* (L.) Calest.). This plant was previously considered a *Cachrys* species but, at present, it is part of the *Prangos* genus. *P. ferulacea* is an orophilous plant present in the eastern Mediterranean and in western Asia. Three different extraction techniques were utilized. Obtained extracts were compared both for their phytochemical content and for their photobiological properties on human melanoma cells irradiated with UVA light. The apoptotic responses, together with the antioxidant activity, were also assessed. *P. ferulacea* extracts were able to affect cell viability in a concentration-dependent manner, with the sample obtained through supercritical CO_2_ extraction showing the highest activity (IC_50_ = 4.91 μg/mL). This research points out the interesting content in the photoactive compounds of this species, namely furanocoumarins, and could provide a starting point for further studies aimed at finding new photosensitizing agents useful in cancer photochemotherapy.

## 1. Introduction

A number of studies concerning the phytochemical composition of plants belonging to the *Cachrys* species highlighted the abundance of coumarins, a family of benzopyrones, and their derivatives, mainly furanocoumarins [1,2,3,4]. The *Cachrys* group (Apiaceae) is divided into several genera, namely *Cachrys*, *Prangos*, *Alocacarpum*, *Bilacunaria*, *Ferulago*, *Diplotaenia*, *Eriocycla*, and *Azilia* [5,6]. Furanocoumarins possess a furan ring fused with the coumarin skeleton. These compounds can be classified into two groups, namely the linear and angular type, according to the attachment place of the furan ring [7].

Beside other biological properties, coumarins and their derivatives have been investigated for their anticancer activity, and it has been demonstrated that their mechanism of action is generally caspase-dependent apoptosis [8]. The potential application of these molecules in the treatment of malignant melanoma has also been assessed [8,9].

Despite significant advances in diagnosis and treatment, skin cancer is one of the leading causes of death and melanoma is the most aggressive form of skin cancer [10]. Beyond the earliest treatment options, such as surgery, chemotherapy, and radiation, more recent therapeutic approaches including nanodrugs, immunotherapy, and photochemotherapy, have been introduced [11]. Photochemotherapy is a very promising approach in anticancer research, in which a photosensitizing agent exerts an antiproliferative effect after interaction with a suitable light, and they exert these effects at doses at which both the photosensitizer and the light alone are not effective [12].

The PUVA (psoralen + UVA) therapy, whose name is an acronym for psoralen plus ultraviolet-A radiation, is a kind of photochemotherapy based on the oral or topical administration of psoralens followed by exposure to UVA radiations [13]. As well as its use in the treatment of psoriasis, vitiligo, and other dermatological diseases, this therapeutic modality is one of the first line options for the treatment of mycosis fungoides, the most common type of cutaneous T-cell lymphoma, which is a heterogenous group of non-Hodgkin lymphomas arising in the skin [14].

In our previous studies focusing on the search for photoactive phytochemicals, we highlighted the biological properties of different plant species belonging to *Cachrys* genus, such as *Cachrys pungens* Jan [15]. The methanolic extract of the aerial parts, together with its chloroform fraction and the coumarins fraction, induced photocytotoxic effects on UVA-irradiated melanoma cells. More recently, we also investigated the photocytotoxic potential of coumarin-rich extracts from *C. sicula* and *C. libanotis* aerial parts, obtained through two extraction methods, namely traditional maceration and pressurized cyclic solid–liquid (PCSL) extraction [16].

In view of these promising previous results, we decided to investigate the photobiological properties of another species, namely *Prangos ferulacea* Lindl. The name is a synonym of *Cachrys ferulacea* (L.) Calest., as this plant, which currently resides in the *Prangos* genus, has been previously considered to be a *Cachrys* species [17]. Indeed, the classification of the genus *Cachrys* has been somewhat confused, and according to recent molecular phylogenetic studies, the genera *Prangos, Cachrys* L., *Azilia*, *Bilacunaria* Pimenov & V.N. Tikhom., *Diplotaenia* Boiss., *Eriocycla*, and *Ferulago* W.D.J.Koch belong to the *Cachrys* clade [18].

*P. ferulacea* is an orophilous species of the eastern Mediterranean and western Asia, being present in Italy, Sicily, the Balkans, Syria, Caucasia, and Iran [19,20]. In these regions, it is also used as animal fodder [21]. This species has been shown to be extremely rich in coumarins, the main class of secondary metabolites detected so far. In addition, the aerial parts also contain several flavonoid glycosides [5]. Shokoohinia and colleagues described the presence of osthole, psoralen, isoimperatorin, oxypeucedanin, oxypeucedanin hydrate, gosferol, oxypeucedanin methnolate, and pranferol in the root extract of the plant [22]. The composition of the essential oil from its fruits has also been described, and γ-terpinene and α-pinene have been described as the major constituents [23].

Different biological properties, such as antimicrobial properties, hypoglycemic activities, and analgesic effects, have previously been demonstrated for this plant [24,25,26]. Bruno and coworkers also reported the antioxidant, cytotoxic, and acetylcholinesterase inhibitory activities of the essential oil from this species, which showed a moderate cytotoxic activity on HCT116 human colon carcinoma, MDA-MB 231 breast carcinoma, and A375 melanoma cell lines [6].

To the best of our knowledge, despite the known content in photoactive compounds (namely furanocoumarins), the photosensitizing properties of *P. ferulacea* have never been investigated so far.

The present study was designed, in particular, to evaluate, for the first time, the photocytotoxic properties of *P. ferulacea*, which were assessed on a human melanoma C32 cell line irradiated with UVA light. Moreover, particular attention was paid to the technique utilized to extracts the photoactive compounds responsible for such activity. To this end, traditional and innovative methods were used and compared. The aerial parts were extracted using three different techniques, namely traditional maceration (TM), supercritical CO_2_ (S-CO_2_), and pressurized cyclic solid–liquid extraction (PCSL) using a Naviglio extractor^®^. These last two extraction procedures have emerged as promising technologies over conventional techniques for the extraction of bioactive compounds from vegetable sources [27,28,29]. Conventional techniques, such as maceration or Soxhlet extraction, require a large quantity of solvent and longer extraction time, and are also accompanied by a low extraction rate and solvent contamination. Because of these shortcomings, above all the use of toxic and/or carcinogenic organic solvents, the interest of the researchers is towards novel, efficient, and green extraction techniques [27].

The rapid solid–liquid dynamic extraction performed using the Naviglio extractor^®^, based on a new solid–liquid extraction principle called Naviglio’s principle, is a technique that is able to reduce extraction times and that allows us to obtain higher yields compared to traditional procedures [30].

A supercritical fluid is a liquid or a gas at atmospheric conditions which is characterized by an interesting dissolving power in its supercritical region, namely when it is heated above its critical temperature and compressed above its critical pressure. Supercritical fluid extraction is a separation process in which the chemical compounds are dissolved in a supercritical fluid, and it is useful to selectively extract a specific compound or fraction from an extract [31].

The phytochemical composition of the *P. ferulacea* extracts was assessed with gas chromatography–mass spectrometry (GC–MS), and principal component analysis (PCA) was used to explore the data patterns and to highlight differences among the composition of *P. ferulacea* extracts. The in vitro photobiological properties were assessed on the melanoma C32 cell line, and the apoptotic responses were also investigated. The in vitro antioxidant activity was assessed as well.

## 2. Materials and Methods

### 2.1. Chemicals

Ascorbic acid, DPPH, propyl gallate, β-carotene, linoleic acid, Tween 20, chlorogenic acid, quercetin, Folin–Ciocalteu reagent, RPMI-1640 medium, fetal bovine serum, penicil-lin/streptomycin, L-glutamine, trypan blue, phosphate buffered saline, Hanks’ balanced salt solution, and tetrazolium salt MTT were supplied by Sigma-Aldrich S.p.a. (Milano, Italy). Melanoma C32 cells were obtained from Type Culture Collection (ATCC) no. CRL-1585. Cyclin D1, p53 p21, PARP, GAPDH, and peroxidase-coupled goat anti-mouse or goat anti-rabbit antibodies were obtained from Santa Cruz Biotechnology (Heidelberg, Germany); ECL System (Amersham Pharmacia Biotech, Cologno Monzese, Italy). All other reagents were obtained from VWR International s.r.l. (Milan, Italy).

### 2.2. Preparation of P. ferulacea Extracts

*P. ferulacea* (syn. *C. ferulacea* (L.) Calest.) aerial parts were collected in Calabria (Southern Italy). A voucher specimen (leg. det. Carmine Lupia) was deposited at the Mediterranean Etnobotanical Conservatory, Sersale, Catanzaro (position number 35 of the Apiaceae section). Plant material was air-dried at room temperature and extracted with methanol through both the maceration technique (72 h × 3 times, plant-to-solvent ratio 1:10 g/mL) and a Naviglio extractor^®^ (Atlas Filtri SrL, Limena, PD, Italy), using a plant:solvent ratio 1:10 g/mL × 2 cycles [32]. Both obtained total extracts were dried under reduced pressure at 40 °C, weighed, and stored at 4 °C until the analyses took place.

A further extract from *P. ferulacea* aerial parts was also obtained through supercritical CO_2_ extraction. About 20 ± 2 g of *P. ferulacea* were placed in the vessel extractor. Supercritical CO_2_ extraction experiments were carried out in a laboratory-scale plant (Spe-ed SFE Applied Separations, USA) using a temperature of 40 °C and a pressure of 250 bar. The carbon dioxide used (purity > 99.99%) was supplied by SIAD Spa (Bergamo, Italy), the extraction time was of about 3 h, and the extractor worked discontinuously; static phases of 15 min were alternated to dynamic extraction phases of the same duration to improve the yield [33].

### 2.3. GC-MS Analyses 

The apolar constituents, namely the coumarins, terpenes, and fatty acids, present in the three different *P. ferulacea* extracts were identified through gas chromatography–mass spectrometry (GC–MS). Analyses were performed using a Hewlett-Packard 6890 GC connected to a Hewlett-Packard 5973 selective mass detector and equipped with an SE-30 (100% dimethylpolysiloxane) capillary column (30 m × 0.25 mm, 0.25 μm film thickness). Analyses were carried out using helium as the carrier gas (linear velocity 0.00167 cm/s) and a programmed temperature from 60 to 280 °C (with a rate of 16 °C /min). The column inlet was set at 250 °C. The MS operating parameters were as follows: ion source, 70 eV; ion source temperature, 230 °C; electron current, 34.6 μA; vacuum 10–5 torr. Mass spectra were acquired over a 40–800 amu range at 1 scan/s. Constituents were identified by comparison of their GC retention times with those of available standards, and by comparing their mass spectra with those from Wiley Mass Spectral Database of the GC–MS system [34]. 

### 2.4. DPPH Assay

The radical scavenging activity of *P. ferulacea* extracts was assessed using the well-established DPPH test, performed as previously described [35]. Briefly, 800 µL of a 0.1 mM methanolic solution of the radical 2,2-diphenyl-1-picrylhidrazyl (DPPH) were mixed with 200 µL of each sample (concentrations ranging from 5 to 1000 µg/mL). Ascorbic acid was used in the positive control group and absorbance was measured at 517 nm after 30 min of incubation in the dark. 

### 2.5. β-Carotene Bleaching Assay

The antioxidant activity of *P. ferulacea* extracts was also estimated using the β-carotene-linoleate bleaching method, as previously described [34]. One milliliter of a 0.2 mg/mL chloroform solution of β-carotene were added to linoleic acid (0.02 mL) and 100% Tween 20 (0.2 mL). The solvent was then evaporated, and distilled water (100 mL) was added. A total of 5 mL of the resulting emulsion was transferred into tubes containing 0.2 mL of each sample (concentration range 0.25–100 µg/mL). The mixtures were placed in a water bath at 45 °C, and the oxidation of the emulsion was monitored by measuring the absorbance at 470 nm over a 60 min period, namely at the initial time and after 30 and 60 min. The same procedure was followed with propyl gallate, which was used as a positive control. The antioxidant activity was calculated in terms of the successful prevention of β-carotene bleaching.

### 2.6. Evaluation of In Vitro Photocytotoxic Effects

The photocytotoxic effects of *P. ferulacea* extracts were assessed in vitro on a UVA-irradiated human C32 melanoma cell line. Cells were cultured in RPMI-1640 medium supplemented with 10% FBS, 1% L-glutamine, and 1% penicillin/streptomycin at 37 °C in a humidified atmosphere of 5% CO_2_ until a monolayer was formed, avoiding the phase of cells overlapping. The photocytotoxicity was evaluated as previously reported [36]. Briefly, 3.8 × 10^4^ cells were placed in each well of a 96-well tissue culture microtiter plate (100 µL/well). Twenty-four hours later, the medium was removed. The *C. ferulacea* extracts were dissolved in MeOH (final solvent concentration 0.5% *v*/*v*), diluted with Hanks’ balanced salt solution (HBSS, pH 7.2), and added to the microtiter plates (100 µL/well) in order to have final concentrations ranging from 0.625 to 100 µg/mL. After 30 min, the plates were irradiated at 365 nm for 1 h at a dose of 1.08 J/cm^2^ using a HPW 125 Philips lamp. The spectral irradiance of the source (0.3 mW/cm^2^) was measured with a radiometer equipped with a 365-CX sensor (Cole-Parmer Instrument Company, Niles, IL, USA). Sample solutions were then replaced with fresh medium, and microtiter plates were incubated for a further 48 h. Bergapten was used as a positive control, and experiments were performed in quadruplicate.

The cytotoxic activity was assessed 48 h later with the 3-[4,5-dimethyl-2-yl]-2,5-diphenyl tetrazolium bromide (MTT) assay, as previously reported [37]. Briefly, medium was removed and 0.5% *w*/*v* MTT (100 μL/well) was added. After 4 h of incubation, DMSO (100 μL/well) was added to dissolve the formazan crystals and absorbance was measured at 550 nm by means of a microplate reader (Stat fax 3200, Awareness Technology Inc., Palm City, FL, USA).

Cell morphology was visualized by using an inverted microscope (AE20 Motic, Motic Instruments, Inc., VWR, Milano, Italy), and photos were captured with a digital camera (VisiCam 3.0 USB, VWR, Milano, Italy).

### 2.7. Immunoblotting Analysis

The melanoma cells (C32) were harvested and lysed in 500 µL of RIPA buffer for total protein extraction. Here, 8% SDS-polyacrylamide gel was used for the resolution of the proteins that were transferred to a nitrocellulose membrane. Then, the filter was probed with cyclin D1, p53, p21, PARP, and GAPDH antibodies (Santa Cruz, Biotechnology, Heidelberg, Germany). The antibody antigen complex was detected with a secondary antibody conjugated to horseradish peroxidase and revealed with the ECL System (Amersham Pharmacia Biotech, Cologno Monzese, Italy) [38].

### 2.8. Statistical Analysis

Three independent measurements were carried out for phytochemical analyses. Biological assays were performed in quadruplicate, and data were expressed as mean ± S.E.M. D’Agostino–Pearson’s K2 test and Levene’s test were used to check the normality of data and homogeneity of variances. For calculation of the half inhibition concentration (IC_50_), raw data were fitted through nonlinear regression in Graph Pad Prism 5 (Graph Pad Software Inc., San Diego, CA, USA), and curves were plotted using the equation log (inhibitor) vs. response—variable slope. Statistical differences between the control and treated groups and between treated group means were tested by one-way analysis of variance (ANOVA), followed by Dunnett’s multiple comparison test and a Bonferroni post-hoc test, respectively (*p* < 0.05, Sigma Stat Software, Jantel Scientific Software, San Rafael, CA, USA). 

Principal component analysis (PCA) was performed with the online software MetaboAnalyst version 5.0 (http://www.metaboanalyst.ca, accessed on 27 October 2022). Data were checked for integrity, and zero values were replaced with small values (1/5 of the minimum positive value for each variable found within the original data). Data were then pretreated through Pareto-scaling. The phytochemical compounds extracted through the three different extraction techniques were graphed through a clustered heat mapping technique, using MetaboAnalyst version 5.0.

## 3. Results 

### 3.1. Phytochemical Constituents

The aerial parts from *P. ferulacea* were collected in Calabria (southern Italy) and extracted with methanol through traditional maceration (TM) and pressurized cyclic solid–liquid (PCSL) extraction using a Naviglio extractor^®^. A further sample was obtained through supercritical CO_2_ extraction (S-CO_2_). The TM technique allowed us to obtain a higher yield (14.7%) compared to the other two methods (3.6% for PCSL and 2.4% with S-CO_2_, Table 1).

The main aim of the phytochemical investigation was the identification of furanocoumarins, the class of compounds responsible for the photosensitizing effects. To this end, gas chromatography–mass spectrometry was performed. The analyses allowed us to identify coumarins and other chemical constituents (Table 2).

Five furanocoumarins were detected: psoralen, xanthotoxin, bergapten, isopimpinellin, and marmesin. These constituents were found in higher number and percentages in the extract obtained through supercritical fluid extraction compared to the other ones. This last technique allowed the extraction of marmesin, which is a precursor in furanocoumarin biosynthesis [39], and psoralen, which were not identified in the other two extracts. Xanthotoxin, bergapten, and isopimpinellin, also present in other extracts, were more abundant, with percentages equal to 3.14%, 4.30%, and 2.17%, respectively. The same trend was observed for the extraction of simple coumarins, as follows: the use of supercritical carbon dioxide (S-CO_2_) allowed the extraction of three compounds, namely citropten, isomeranzin, and osthole, while only the latter molecule was identified, at a lesser extent, in the other two *P. ferulacea* extracts (TM and PCSL). Moreover, four terpenes, namely estragole, trans-caryophyllene and neophytadiene, together with a number of fatty acids, were also recognized. These two last classes of phytochemicals were more abundant in the TM sample. With regard to the fatty acid composition, the highest percentages were observed for palmitic acid (8.49%), 7,10,13-hexadecatrienoic acid (4.62%), lignoceric acid (4.39%), and myristic acid (3.24%).

**Figure 1:**
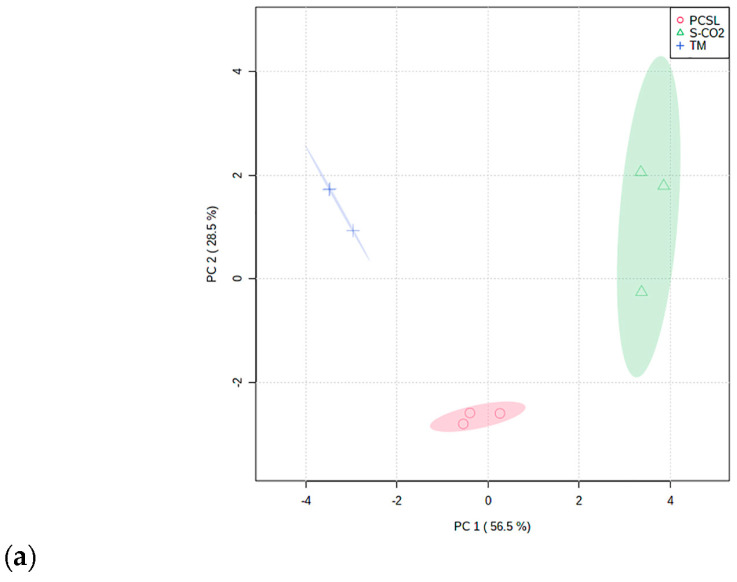

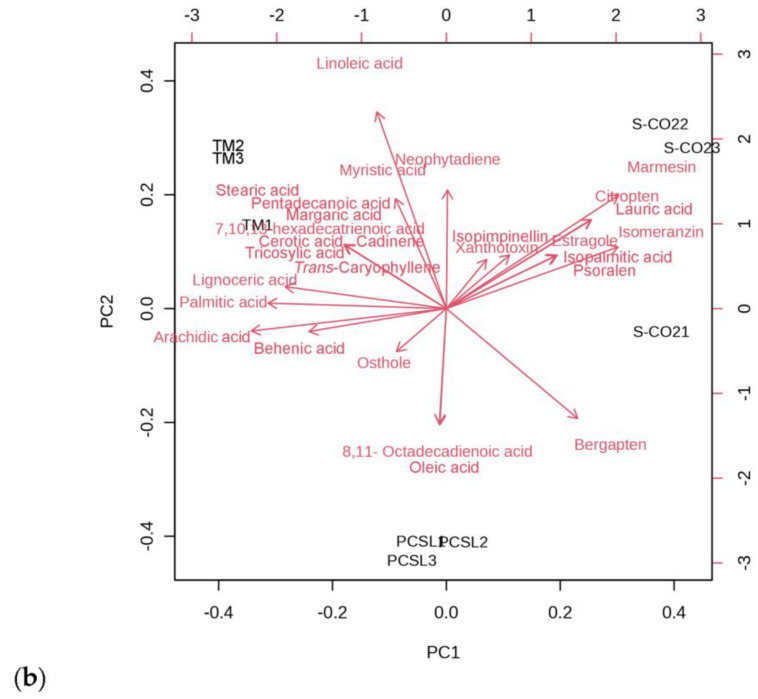
Scores plot (**a**) and loadings bi-plot (PC-1 vs. PC-2) (**b**) of *P. ferulacea* extracts’ phytochemical profile. Abbreviations are as follows: TM, traditional maceration; PCSL, pressurized cyclic solid–liquid extraction, S-CO_2_: supercritical CO_2_ extraction.

A clear overview of the distribution of identified metabolites in the three extracts was evidenced by principal component analysis. Figure 1 describes the scores plot (a) and the biplot of scores and loadings values (b), by considering the first and the second principal components, with a total explained variance of 85%. 

The S-CO_2_ samples, located in the top right half of the scores and loadings biplot, were characterized by the highest content of coumarins and furanocoumarins. The TM samples were located in the top left half of the biplot, which could be explained as being due to their higher amounts of fatty acids compared to the other samples. 

**Figure 2:**
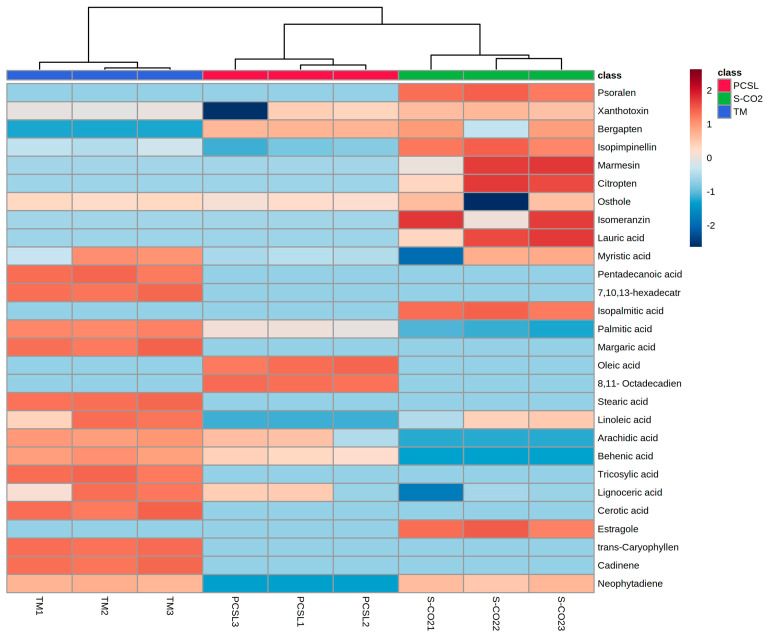
Heatmap of identified phytochemicals. Abbreviations are as follows: TM, traditional maceration; PCSL, pressurized cyclic solid–liquid extraction; S-CO_2_, supercritical CO_2_ extraction.

These differences in the relative content of significant discriminant secondary metabolites are also visualized in the heatmap reported in Figure 2.

### 3.2. Antioxidant Potential

**Figure 3:**
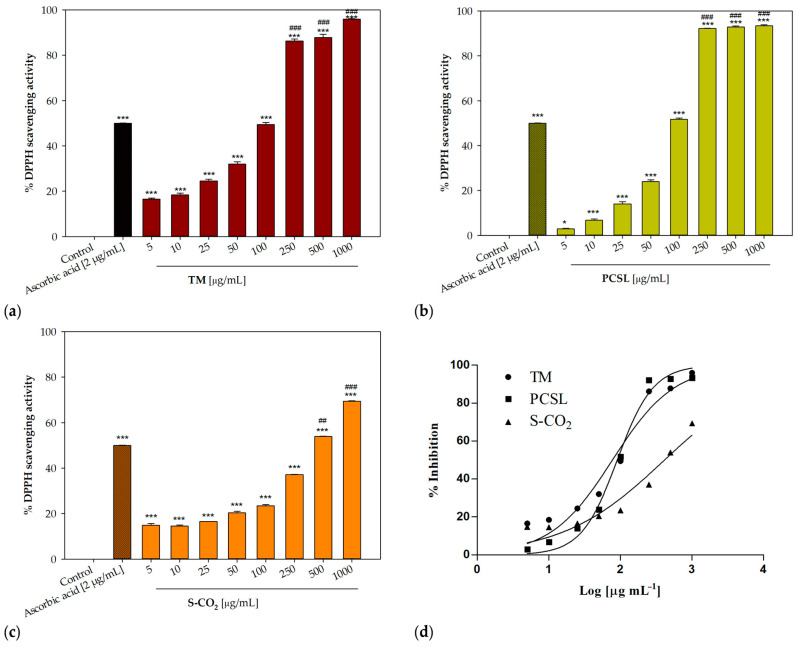
Concentration-dependent radical scavenging activity of *P. ferulacea* Lindl. extracts. (**a**) Traditional maceration, TM; (**b**) pressurized cyclic solid–liquid extraction, PCSL; (**c**) supercritical CO_2_ extraction, S-CO_2_; (**d**) nonlinear regression analyses; * *p* < 0.05, *** *p* < 0.001 compared to control (Dunnett’s test); ## *p* < 0.01 ### *p* < 0.001 compared to positive control (ascorbic acid, 2 µg/mL).

**Figure 4:**
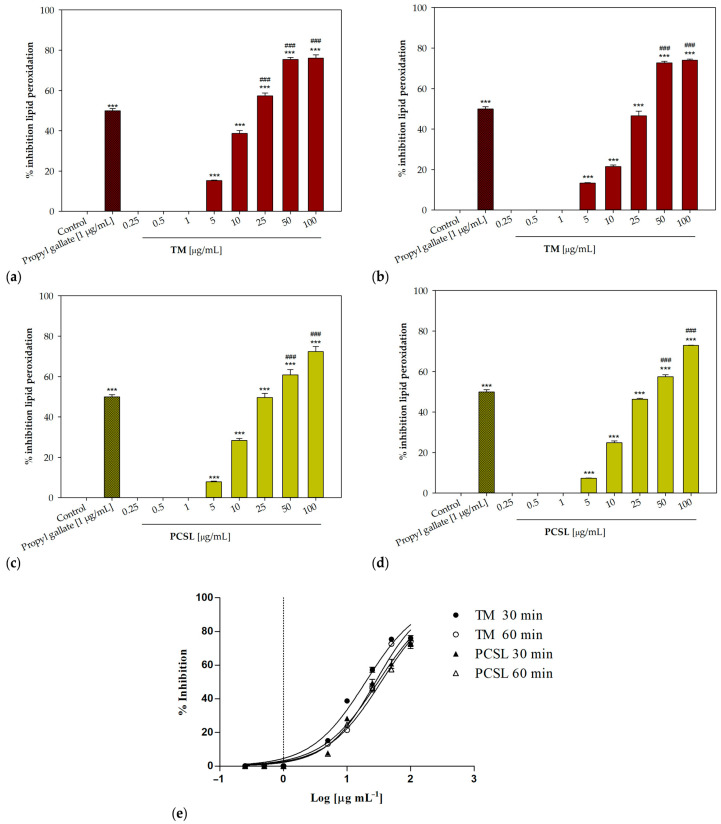
Concentration-dependent inhibition of lipid peroxidation induced by *P. ferulacea* Lindl. extracts. (**a**) Traditional maceration, TM, 30 min of incubation; (**b**) traditional maceration, TM, 60 min; (**c**) pressurized cyclic solid–liquid extraction, PCSL, 30 min.; (**d**): pressurized cyclic solid–liquid extraction, PCSL, 60 min; (**e**) nonlinear regression analyses; *** *p* < 0.001 compared to control (Dunnett’s test); ### *p* < 0.001 compared to positive control (propyl gallate, 1 µg/mL).

The in vitro antioxidant potential of *P. ferulacea* extracts was assessed using the DPPH radical scavenging assay and the β-carotene bleaching test. Ascorbic acid [40] and propyl gallate [41,42] were used as positive controls, respectively. All three *P. ferulacea* extracts induced a concentration-dependent radical scavenging activity (Table 3, Figure 3). The sample obtained with traditional maceration showed the best radical scavenging potency (IC_50_ = 77.37 ± 1.58 μg/mL). A better antioxidant activity was also observed in the second test, with IC_50_ values equal to 19.57 ± 0.67 and 27.94 ± 0.48 μg/mL after 30 and 60 min of incubation, respectively (Figure 4).

The sample extracted with the Naviglio extractor^®^ (PCSL) was also effective in protecting linoleic acid from peroxidation, even if only to a minor extent, with IC_50_ values equal to 30.75 ± 1.11 and 34.27 ± 0.35 μg/mL after 30 and 60 min, respectively.

### 3.3. Photocytotoxicity

The photocytotoxic properties of investigated samples were evaluated on a melanoma C32 cell line. Cell cultures were treated with different concentrations of each sample (ranging from 0.625 to 100 μg/mL), dissolved in MeOH and diluted with Hanks’ balanced salt solution (HBSS, pH 7.2), in 96-well tissue culture microtiter plates. Plates were incubated at 37 °C for 30 min and then irradiated with UVA light for 1 h at a dose of 1.08 J/cm^2^. All three *P. ferulacea* extracts affected cell viability in a concentration-dependent manner. Extracts obtained through TM and PCSL extraction showed a good biological activity, with IC_50_ values equal to 27.95 ± 0.67 and 25.90 ± 1.23 μg/mL. Consistent with the highest furanocoumarin abundance, the best activity was observed for the extract obtained through supercritical CO_2_ extraction, with an IC_50_ of 4.91 ± 0.15 μg/mL (Table 4).

**Figure 5:**
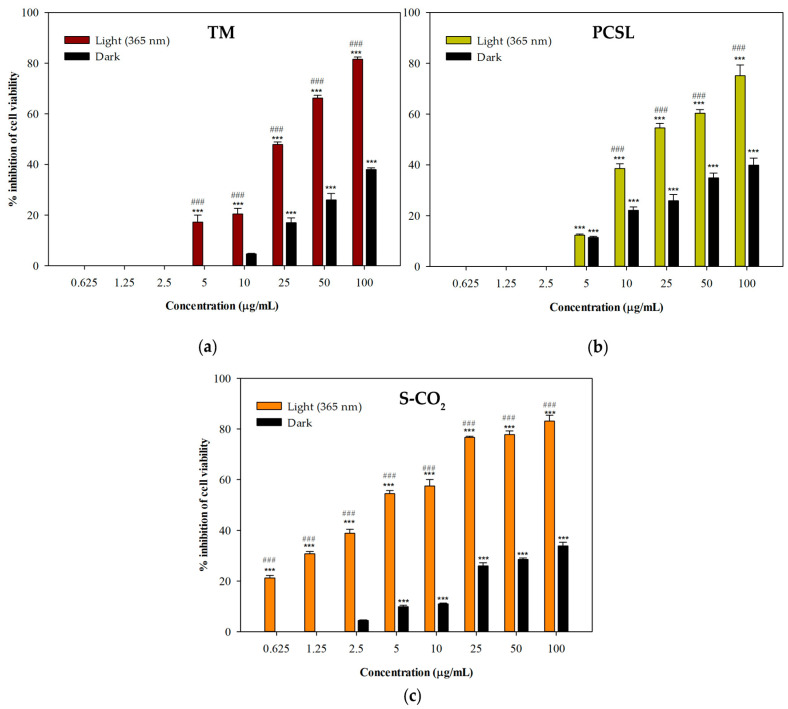
Concentration-dependent photocytotoxic effects and dark toxicity of *P. ferulacea* Lindl. extracts. (**a**) Traditional maceration, TM; (**b**) pressurized cyclic solid–liquid extraction (PCSL); (**c**) supercritical CO_2_ extraction (S-CO_2_); *** *p* < 0.001 compared to control (Dunnett’s test); ### *p* < 0.001 compared to cytotoxic effects in the dark.

Interestingly, no samples were significantly cytotoxic in the dark. At all the concentrations tested, the extracts showed enhanced cytotoxicity upon exposure to UV light, as illustrated (Figure 5). This is very interesting, as a proper photosensitizing agent should not have high levels of dark toxicity [43,44].

**Figure 6:**
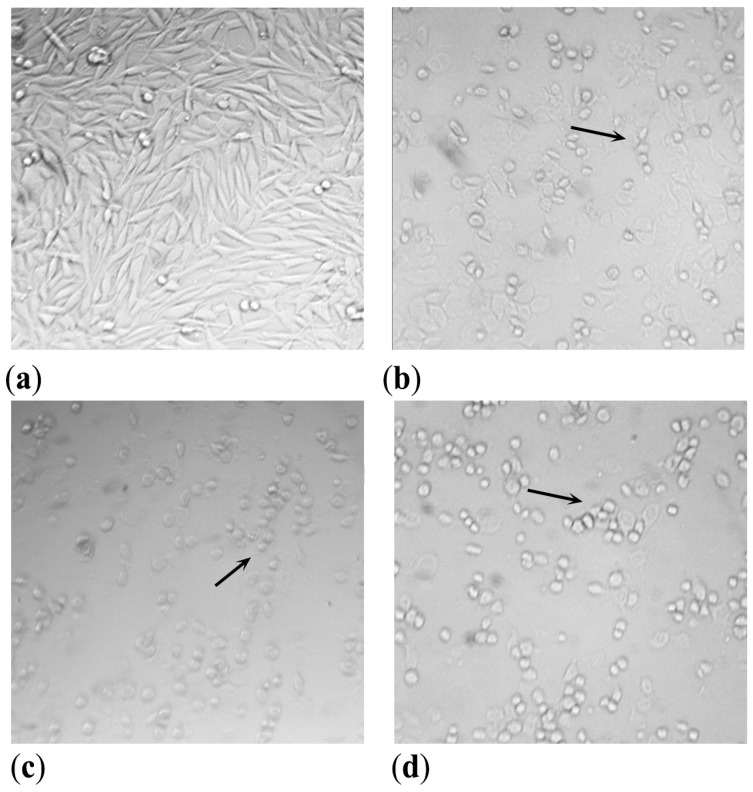
Melanoma C32 cells morphology 48 h after UVA irradiation at a dose of 1.08 J/cm^2^. (**a**) Control, untreated irradiated cells in RPMI 0.5% MeOH; (**b**) treated irradiated cells, *P. ferulacea* extracted with maceration (TM), 50 µg/mL; (**c**) treated irradiated cells, *P. ferulacea* extracted with Naviglio^®^ (PCSL), 50 µg/mL; (**d**) treated irradiated cells, *P. ferulacea* L. extracted with supercritical CO_2_ extraction (S-CO_2_), 25 µg/mL. Cells were visualized with an inverted microscope (AE20 Motic) and images were captured with a VisiCam digital camera. Arrows indicate rounded and shrunken cells. Magnification, 10×.

The cell morphology 48 h after treatment and UVA irradiation was captured on a digital camera (Figure 6). 

As is shown, the incubation of cell cultures with the TM and PCSL samples at a concentration of 50 µg/mL strongly affected cell viability compared to the control. Even better results were observed for the sample obtained with supercritical carbon dioxide; as reported in Figure 4c, cells treated with S-CO_2_ extract became rounded and shrunken even at a lower concentration of 25 µg/mL.

### 3.4. Apoptotic Responses

**Figure 7:**
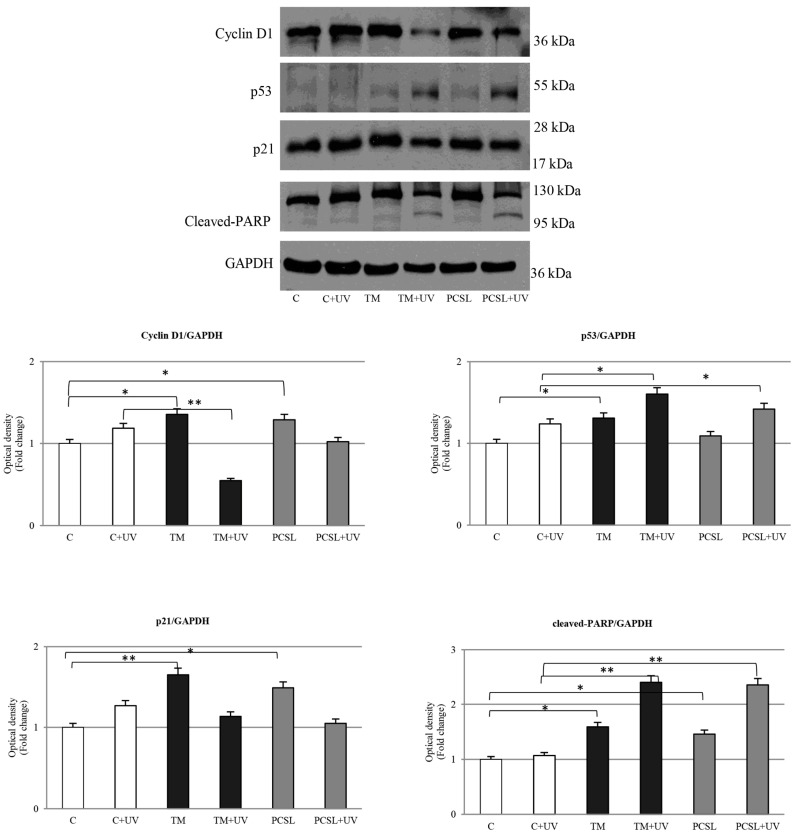
Western blot analysis of Cyclin D1, p53, p21, and PARP (poly ADP-ribose polymerase) protein levels in C32 cells treated or not with TM and PCSL extracts for 24 h, both in the presence and absence of UV. The histograms represent the mean ± SD of three separate experiments in which band intensities were evaluated as optical density (OD) and expressed as fold change relative to control; * *p* ≤ 0.05; ** *p* ≤ 0.005 vs. control (C) and control + UV (C+UV).

**Figure 8:**
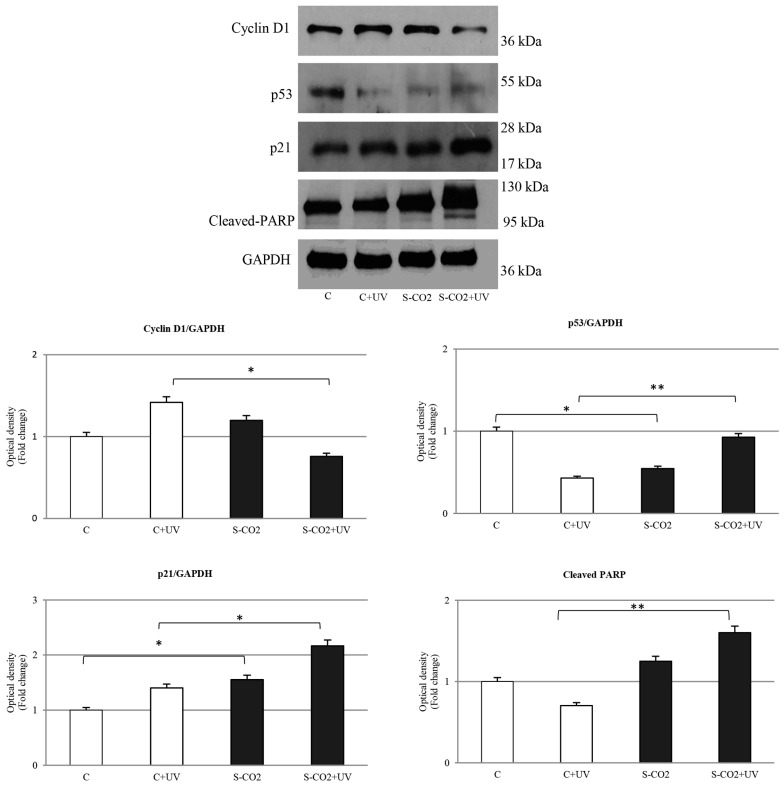
Western blot analysis of Cyclin D1, p53, p21, and PARP (poly ADP-ribose polymerase) protein levels in C32 cells treated or not with S-CO_2_ extract for 24 h, both in the presence and absence of UV. The histograms represent the mean ± SD of three separate experiments in which band intensities were evaluated as optical density (OD) and expressed as fold change relative to control; * *p* ≤ 0.05; ** *p* ≤ 0.005 vs. control (C) and control + UV (C+UV).

Immunoblotting analysis has evidenced a downregulation in Cyclin D1 expression in C32 UV-treated cells and an increase in the cyclin-dependent kinase inhibitor p21^cip1/waf1^, which results in the cells being subjected to S-CO_2_ extract more consistently (Figure 7 and Figure 8). 

The UVA irradiation of the melanoma cells, treated with extracts obtained through the TM, PCLS, and S-CO_2_ methods, has shown an upregulation in the onco-suppressor p53 protein with respect to the UVA-control cells. 

In addition, we observed an upregulation in the proteolytic form di poly ADP ribose polymerase (PARP), involved in DNA repair mechanisms, in melanoma cells which underwent UVA light treatment (Figure 7 and Figure 8).

## 4. Discussion

The *Prangos* Lindl. genus is comprised of about 45 species distributed from Portugal to Tibet, with the center of the diversity located in the Irano-Turanian region. Phylogenetic studies demonstrated that this genus is related to *Binacularia* and *Cachrys* genera [26], and both *Prangos* and *Binacularia*, together with *Alocarpum*, *Ferulago*, *Diplotaenia Azilia*, and *Eriocycla* are currently members of the so-called *Cachrys* group [5,6].

Both the *Prangos* and *Cachrys* genera are rich in coumarins and particularly in furanocoumarins [26,45].

Coumarins (1,2-benzopyrones or 2H-1-benzopyran-2-ones) consist of a benzene ring linked to the pyrone ring. These benzopyrone compounds are divided into six groups, namely simple coumarins, bicoumarins, furanocumarins, pyranocoumarins, benzocoumarins, and coumestans [46]. Furanocoumarins are tricyclic aromatic derivatives which bear a furan ring fused to the α-benzopyrone skeleton, and can be structurally divided into angular and linear compounds, with the furan ring attached at the 6, 7 or to the 7, 8 position on the aromatic ring (angelicins and psoralens, respectively). Furanocoumarins have interesting photosensitizing properties [47]. Photochemotherapy is a very promising approach in oncology, and is based on the action of a photosensitizing agent which exerts an antiproliferative effect after interaction with a suitable light, and they both exert these effects at doses at which either the photosensitizer and the light alone are not effective [11]. Psoralens, when activated by UV light, are able to interact with DNA, forming cross-links between adjacent strands and, hence, interfering with cellular replication [48]. This kind of photochemotherapy, based on the administration of psoralens followed by exposure to UVA radiation, is called PUVA therapy, and it is used in the treatment of both dermatological diseases (such as psoriasis and vitiligo), and mycosis fungoides, the most common type of cutaneous T-cell lymphoma, which is a heterogenous group of non-Hodgkin lymphomas arising in the skin [14,49].

*Prangos ferulacea* Lindl. (syn. *Cachrys ferulacea* (L.) Calest.) is one of the most studied species previously included in the *Cachrys* genus and currently considered a *Prangos* species [45]. In our study, this plant was tested for the first time for its photobiological properties and for its potential use in the treatment of melanoma. Moreover, the aerial parts of the plant were extracted using three different extraction techniques, both traditional methods (maceration), and innovative ones, namely rapid solid–liquid extraction dynamic extraction via the Naviglio extractor, and supercritical CO_2_ extraction. Even if the traditional maceration allowed us to obtain the highest extraction yield, furanocoumarins were detected in higher number and percentages in the extract obtained through supercritical fluid extraction. This last extract showed the presence of xanthotoxin, bergapten, and isopimpinellin, together with other two compounds, namely marmesin and psoralen, which were found in this sample only. The same extraction method also allowed us to obtain a higher abundance of simple coumarins (citropten, osthole, and isomeranzin) compared to the other two. Some terpenes were detected as well.

These classes of phytochemicals were previously reported by Bertoli and colleagues, who investigated the phytochemical composition of the essential oils from the different parts of *P. ferulacea*, reporting the presence of the terpenes α-pinene, sabinene, and limonene, as well as the coumarin osthole [50]. Pistelli and coworkers reported the presence of osthol, imperatorin, and isoimperatorin, oxypeucedanin, decursin, and heraclenin in the root and the seed of the plant [3]. Moreover, the compounds osthol, bergapten, imperatorin, and isoimperatorin were described in the extract from fruits from Sicily extracted with ethyl acetate with Soxhlet [51], and Badalamenti and colleagues identified a new chemotype, characterized by a large amount of (Z)-β-ocimene, from Sicily [52].

Our study evidenced the influence of the utilized extraction method on the phytochemical composition of obtained extracts. As evidenced by principal component analysis, the S-CO_2_ samples were characterized by the highest content of coumarins and furanocoumarins, while the TM samples showed a higher amount of fatty acids compared to the other samples.

The maceration technique was the best method with regard to the antioxidant activity of *P. ferulacea* samples, which was assessed with two in vitro assays, the DPPH test and the β-carotene bleaching test. Our results are in accordance with other studies which pointed out the antioxidant potential of this species. The essential oils from the leaves and flowers of *P. ferulacea* were proven to induce a decrease in ROS and an increase in the activity of superoxide dismutase (SOD), catalase (CAT), and glutathione S-transferase (GST) in opsonized zymosan-stimulated human polymorphonuclear cells (PMNs) [52]. A good radical scavenging ability was also described by Bagherifar and colleagues [53].

To the best of our knowledge, our study provides the first results as regards the photobiological properties of this species. Here, C32 melanoma cancer cells were treated with different concentrations of each *P. ferulacea* extract, and plates were then irradiated with UVA light at a dose of 1.08 J/cm^2^. All three samples were able to affect cell viability in a concentration-dependent manner. Consistent with the highest furanocoumarin content, the extract obtained through supercritical CO_2_ extraction showed the best activity, with an IC_50_ value equal to 4.91 ± 0.15 μg/mL, without showing a significant cytotoxicity in the dark. This makes *P. ferulacea* extract a very interesting phytocomplex and a potential candidate for further studies, as a proper photosensitizing agent should not have high levels of dark toxicity [40,41].

Malignant melanoma is characterized by high mortality, drug resistance, and metastases. The therapeutic treatments mainly used in the melanoma are surgical resection, immunotherapy, biochemotherapy, and photodynamic and targeted therapy. However, these approaches are not very successful over time, due to the onset of side effects and resistance. For this reason, novel candidate therapeutics are needed. The focus is on natural products, mainly used in traditional medicine for the treatment of various inflammatory diseases, and which have shown fewer side effects [16].

In the present study, our results reveal that the effects of *C. ferulacea* extracts in melanoma cells were accompanied by modulation of the key cell cycle regulatory proteins, namely cyclin D1, p53, and p21^Cip/Waf1^. In fact, it is well known how cancer development and progression are caused by the perturbation of the cell cycle regulatory mechanisms [54].

The cell cycle depends on the activation and inactivation of different cyclin-dependent kinases related to cyclins and cdk-inhibitory sub-unity, such as p21^Cip/Waf1^, that belongs to the Cip/Kip family [55].

In particular, the p21 and p53 proteins increased as a result of DNA damage, resulting in cell cycle arrest and in the modulation of apoptotic responses. As reported by other authors, p21 can inhibit some proteins involved in the activation of apoptosis, such as procaspase 3, and caspases 8 and 10 [54].

In the presence of certain stressors, p21 can induce intrinsic and extrinsic pathways of apoptosis, with an increase in the proapoptotic protein Bax and activation of the tumor necrosis factor (TNF) family of death receptors, respectively [54,56].

In C32 cells which underwent exposure to UVA light, in addition to the upregulation in p21, our results showed an increase in PARP cleavage, which plays key roles in DNA repair, chromatin modulation, and transcription [57]. In conclusion, this study demonstrated that *P. ferulacea* extracts, mainly the S-CO_2_ sample, contain important photoactive constituents responsible for their photocytotoxic activity. The investigated samples induced promising cytotoxic effects on malignant melanoma cells upon irradiation with UVA light, without affecting cell viability in the dark. Future studies could be useful to further optimize the extraction method and to continue investigating the interesting photobiological properties of this species.

## Figures and Tables

**Table 1:** *Prangos ferulacea* Lindl. extracts.

Extraction Technique	Abbreviation	Yield (%)
Maceration	TM	14.7
Naviglio^®^	PCSL	3.6
Supercritical CO_2_	S-CO_2_	2.4

**Table 2:** Phytochemical profile of *Prangos ferulacea* Lindl. extracts.

Compound	RT ^1^	RAP ^2^		
		TM	PCSL	S-CO_2_
Furanocoumarins				
Psoralen	17.645	-	-	2.93 ± 0.25
Xanthotoxin	19.251	1.91 ± 0.04	2.73 ± 0.17	3.14 ± 0.11
Bergapten	19.411	-	2.83 ± 0.13	4.30 ± 0.09
Isopimpinellin	20.645	1.13 ± 0.05	0.89 ± 0.07	2.17 ± 0.14
Marmesin	21.223	-	-	3.96 ± 0.19
Coumarins				
Citropten	18.782	-	-	2.48 ± 0.26
Osthole	19.891	2.42 ± 0.12	2.03 ± 0.19	3.82 ± 0.19
Isomeranzin	20.582	-	-	1.90 ± 0.11
Terpenes				
Estragole	11.192	-	-	0.15 ± 0.02
*trans*-Caryophyllene	13.827	0.81 ± 0.03	-	-
Cadinene	14.816	0.54 ± 0.03	-	-
Neophytadiene	17.450	0.78 ± 0.04	-	0.71 ± 0.06
Fatty acids				
Lauric acid	15.039	-	-	0.10 ± 0.01
Myristic acid	16.496	3.24 ± 0.20	0.25 ± 0.03	2.04 ± 0.09
Pentadecanoic acid	17.336	0.44 ± 0.03	-	-
7,10,13-Hexadecatrienoic acid	17.959	0.97 ± 0.04	-	-
Isopalmitic acid	18.009	-	-	0.55 ± 0.04
Palmitic acid	18.113	8.49 ± 0.49	1.15 ± 0.10	0.14 ± 0.02
Margaric acid	18.891	0.33 ± 0.03	-	-
Oleic acid	19.091	-	0.36 ± 0.03	-
8,11-Octadecadienoic acid	19.371	-	1.08 ± 0.04	-
Stearic Acid	19.617	0.82 ± 0.03	-	-
Linoleic acid	19.702	1.69 ± 0.13	-	0.20 ± 0.02
Arachidic acid	20.988	1.13 ± 0.08	0.44 ± 0.04	-
Behenic acid	22.263	2.72 ± 0.22	1.33 ± 0.14	-
Tricosylic acid	22.954	1.13 ± 0.07	-	-
Lignoceric acid	23.760	4.39 ± 0.33	0.86 ± 0.04	1.00 ± 0.10
Cerotic acid	25.829	1.40 ± 0.10	-	-
Total compounds		34.34	13.95	29.59

^1^ Retention time (min). ^2^ Relative peak area percentage (TIC %). Each value is the mean ± S.D. of three independent measurements.

**Table 3:** Antioxidant activity of *P. ferulacea* Lindl. extracts.

Sample	IC_50_ (μg/mL)		
	DPPH	β-Carotene	
		30 min	60 min
TM	77.37 ± 1.58 ^b^	19.57 ± 0.67 ^b^	27.94 ± 0.48 ^c^
PCSL	90.27 ± 1.45 ^c^	30.75 ± 1.11 ^c^	34.27 ± 0.35 ^d^
S-CO_2_	413.10 ± 1.79 ^d^	n.a.	n.a.
Ascorbic acid *	2.00 ± 0.01 ^a^	-	-
Propyl gallate *	-	1.00 ± 0.02 ^a^	1.00 ± 0.02 ^a^

Data were expressed as mean ± S. E. M. (n = 3). Abbreviations are as follows: TM, traditional maceration; PCSL, pressurized cyclic solid–liquid extraction; S-CO_2_, supercritical CO_2_ extraction. Different letters along column (DPPH) or between columns (β-carotene bleaching test) indicate statistically significant differences at *p* < 0.05 (Bonferroni post-hoc test); n.a. = not active; * positive controls. Different letters indicate the statistical differences detected through Bonferroni post-hoc test.

**Table 4:** Photocytotoxic effects of *P. ferulacea* Lindl. extracts on human melanoma cell line C32.

Sample	IC_50_ (μg/mL)	
	Irradiated Cells	Unirradiated Cells
TM	27.95 ± 0.67 ^c^	>100
PCSL	25.90 ± 1.23 ^c^	>100
S-CO2	4.91 ± 0.15 ^b^	>100
Bergapten *	0.191 ± 0.012 ^a^	n.d.

Data were expressed as mean ± SEM (n = 4). Different letters indicate statistically significant differences at *p* < 0.05 (Bonferroni post-hoc test). Abbreviations are as follows: TM, traditional maceration; PCSL, pressurized cyclic solid–liquid extraction; S-CO_2_, supercritical CO_2_ extraction; n.d., not detectable; * positive control.

## Data Availability

The data presented in this study are available in the article.
